# “Bone in the penis” or fasciitis ossificans of the penis – a first time description of a pseudo-tumor at an extraordinary site

**DOI:** 10.1186/s12894-024-01475-y

**Published:** 2024-04-09

**Authors:** Sebastian Lenart, Oskar Koperek, Anke Scharrer, Eva Comperat

**Affiliations:** 1 Department of Urology and Andrology, St. John of God Hospital, Vienna, Austria; 2 Laboratories for pathology Kaserer, Koperek and Beer, Vienna, Austria; 3 Department of Pathology, Medical University Vienna, Vienna, Austria; 4 Department of Urology, Paracelsus Medical University Salzburg, Salzburg, Austria

**Keywords:** Fasciitis ossificans, Penile ossification, Penile cancer

## Abstract

**Background:**

Fasciitis ossificans is a rare subtype of nodular fasciitis, a benign soft tissue tumor with reactive characteristics. Due to its rapid growth, it is often misdiagnosed as a malignant tumor. While fasciitis ossificans commonly originates from the subcutaneous tissue and can appear throughout the body, it may also arise from extraordinary sites.

**Case presentation:**

We report the first-ever documented case of fasciitis ossificans arising from the penis in a male patient who presented with a tumor on the glans penis. The tumor was surgically resected due to suspicion of penile cancer. Initial histopathological analysis led to a misdiagnosis of squamous cell carcinoma. However, pathological consultation ultimately confirmed the diagnosis of fasciitis ossificans of the penis originating from the glans penis by demonstrating ossification.

**Conclusion:**

This case underscores the importance of considering fasciitis ossificans in the differential diagnosis of soft tissue tumors, even in unusual locations such as penile soft tissue.

## Background

Fasciitis ossificans is a pseudotumor of soft tissue, classified as a rare subtype of nodular fasciitis—a common reactive neoplasm of fibroblastic or myofibroblastic origin with an unknown etiology [1]. The presence of ossification in nodular fasciitis confirms the diagnosis of fasciitis ossificans. While it primarily affects adults without sex predilection, cases have been reported in children as well. Common locations include the extremities, head and neck, trunk, as well as the breast’s fascia or muscle. Due to its rapid growth, hypercellularity, and cytologic atypia, fasciitis ossificans is often mistaken for malignant sarcoma [2]. In some instances, it may be associated with medical conditions such as sarcoidosis or fibrodysplasia ossificans progressiva (FOP). Clinically, it typically presents as a growing asymptomatic mass, although tenderness, pain, or bleeding may occur. Surgical excision is considered curative, although recurrence can occur following incomplete excision.

While presentations at unusual sites have been described in case reports, to the best of the author’s knowledge, fasciitis ossificans of the penis has not been previously reported. Herein, we present the first documented case of a 73-year-old man presenting with fasciitis ossificans of the penis.

### Case presentation

A 73-year-old man presented at the outpatient clinic with sudden paraphimosis accompanied by a tumor measuring 2 × 2 cm arising from the glans penis, causing narrowing of the external urethral meatus and complaining about painful swelling of the penis persisting for the last two weeks. He noticed the tumor on the day of presentation, experiencing pain due to acute paraphimosis. A slightly and slow swelling of the entire penis had already commenced a couple of weeks prior to his visit, beginning the day after he underwent a rigid cystoscopy at a urology practice. During this cystoscopy, the patient experienced sudden severe pain and subsequently suffered from gross hematuria. In the following days, the swelling of the penis increased and then stabilized, while the hematuria ceased the day after the traumatic cystoscopy. The patient noticed swelling at that time but no further increase until about two weeks before the consultation in the outpatient clinic. He avoided consultation at the urological practice, where the cystoscopy was performed. The urologist there noted from his cystoscopy only a stenotic external urethral meatus without further findings. The patient had reported him urinary difficulties without pain or hematuria for several weeks before seeking urological consultation. Approximately six months prior to this visit, a routine check-up had been conducted without any abnormalities. His medical history included lower urinary tract symptoms attributed to mild benign prostatic hyperplasia (BPH) and “non-insulin-dependent diabetes mellitus”, for which he did not require treatment.

**Fig. 1:**
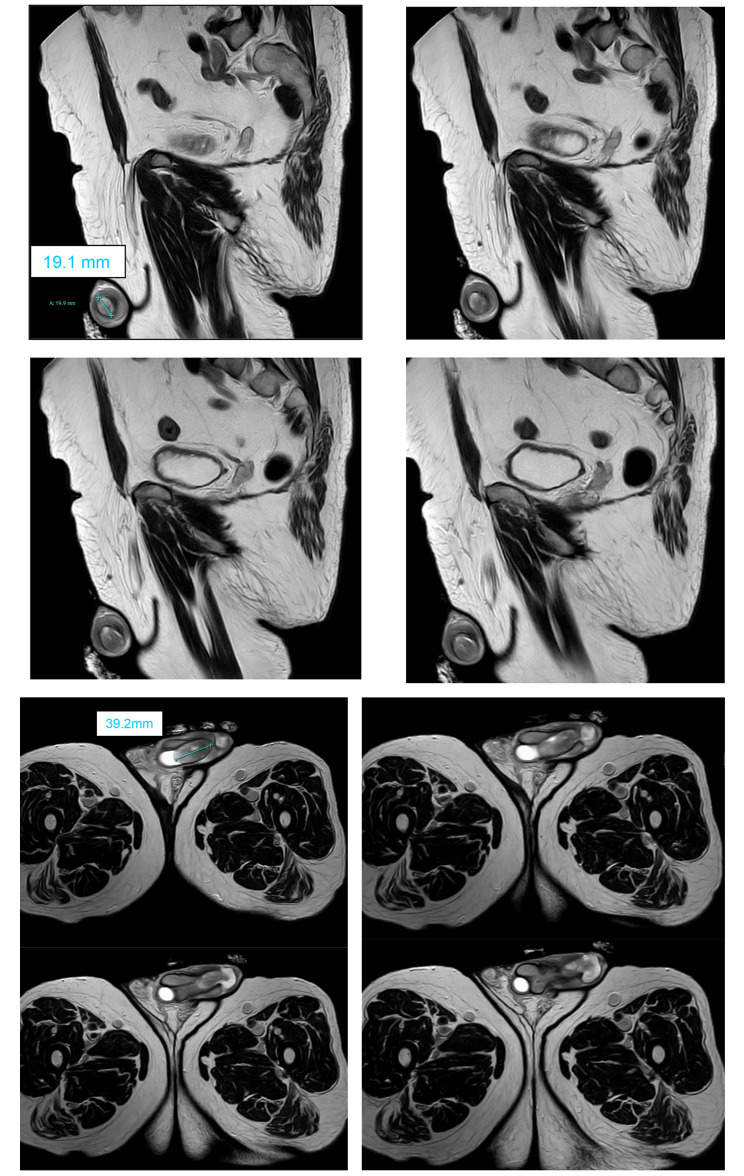
MRI of the tumor formation with size indication

Upon initial presentation at our outpatient clinic, paraphimosis was promptly relieved. Due to suspicion of urethral carcinoma, a biopsy of the tumor was performed. The initial histopathological analysis did not yield definitive results. P-16 analysis was negative, and AE 1/AE 3 stains were not detected. Signs of ossification were already observed. With a provisional diagnosis of “papillary urethritis with metaplastic ossification with high-grade squamous dysplasia—consistent with carcinoma in situ”, the sample was sent for a second opinion. However, this analysis also failed to provide conclusive results due to a high amount of necrotic tissue. Subsequently, MRI imaging was performed, revealing an expansive process in the distal part of the corpus spongiosum measuring 3.8 × 1.9 cm in diameter, with a low apparent diffusion coefficient (ADC) signal alteration and significant contrast media enhancement (Fig. 1). There were no indications of lymph node involvement. After obtaining informed consent from the patient, a partial penile amputation was performed due to suspected penile carcinoma.

**Fig. 2:**
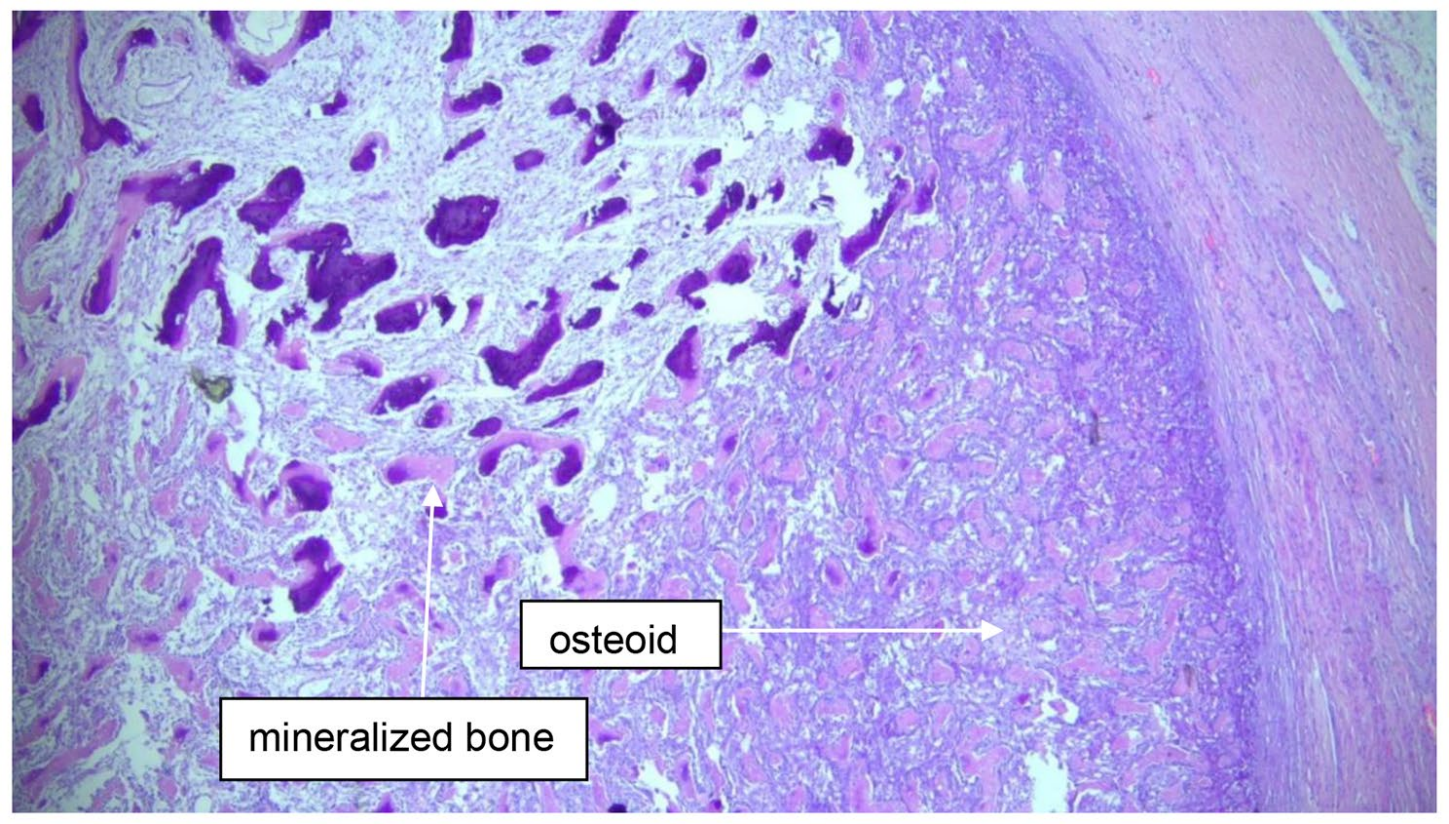
Top left: mineralized bone. Down right: osteoid; magnification 20x; HE (Hematoxylineosin)-staining

**Fig. 3:**
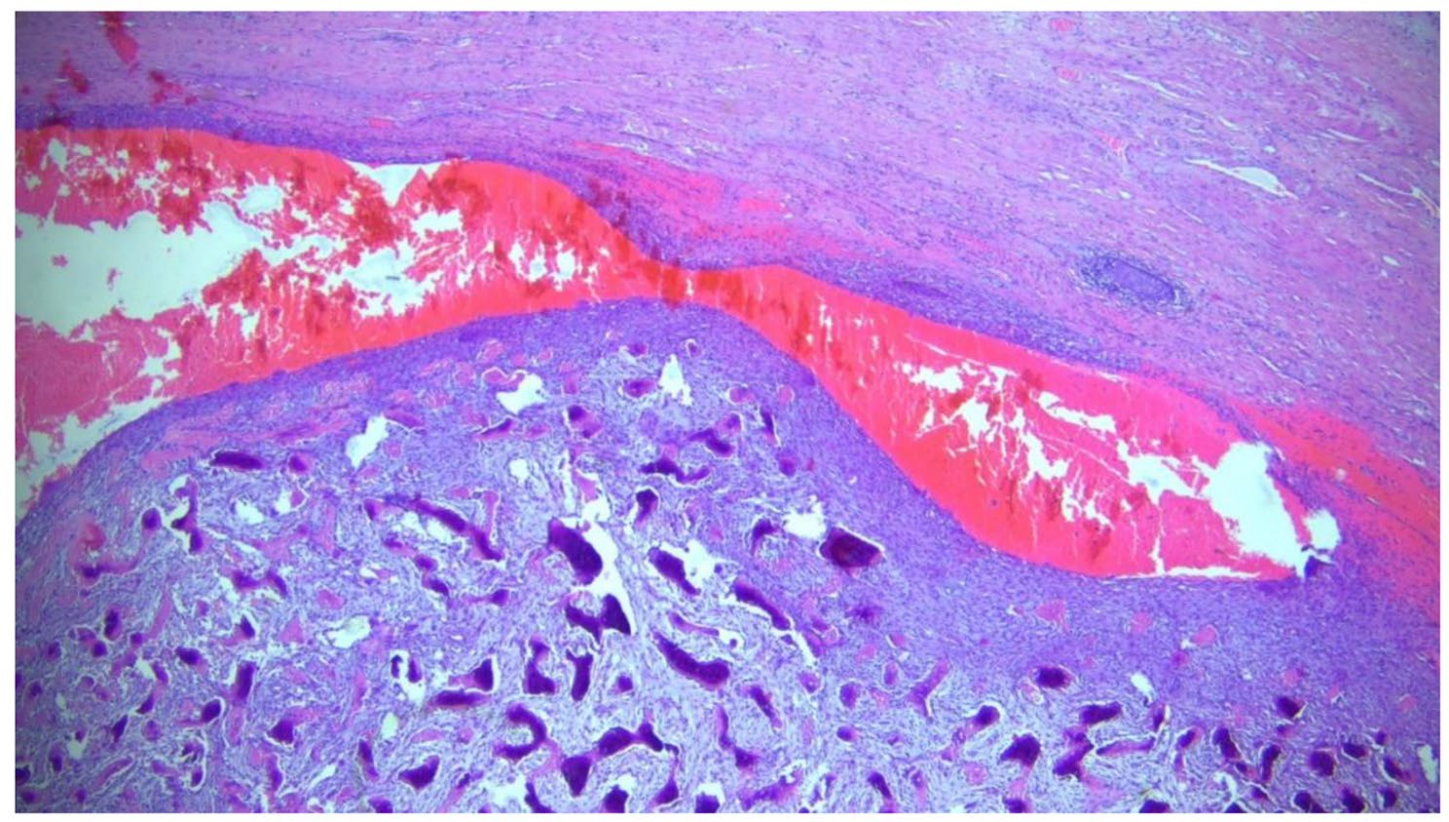
Mineralized bone beside osteoid; magnification 20x; HE (Hematoxylineosin)-staining

Histological examination of the specimen revealed further staining characteristics: desmin negative, smooth muscle actin positive, slightly positive antigen expression of CD10, and CD34 marking vessels. The tumor originated from the glans penis and extended to the urethra without mucosal breach. Mitotic activity was rare and confined to the basal cell layer without p63 hyperexpression, and the Ki-67 proliferation index was low at 10%. Centrally, the tumor exhibited clear signs of ossification, characterized by abundant osteoid, osteoblasts, and osteoclasts (see Figs. 2 and 3). There were no indications of squamous cell carcinoma. Since the mass on the penis was misinterpreted as penile carcinoma at the initial presentation, no photographic documentation was performed.

## Discussion

In this case report, we present a unique instance of fasciitis ossificans occurring in an extraordinary location, which has not been previously documented. Specifically, the tumor originated from the glans penis. The description of this case aims to aid in the diagnostic evaluation of patients with unclear tumor formations of the urethra/penis.

Initially, soft tissue hyperplasia does not exhibit ossification. The development of ossification is a delayed but rapid process, typically occurring 2–6 weeks from onset until clinical presentation. Concurrently, bone tissue maturation occurs. The tumor typically reaches a characteristic macroscopic size of approximately 3 cm on average, but may occasionally grow larger. While often asymptomatic, it can manifest as tenderness, pain, bleeding, or neuropathic sensations. Radiological examination typically reveals a soft tissue tumor with circumscribed growth, without infiltration of underlying tissue. However, imaging findings are nonspecific and may pose challenges in differentiation from sarcoma. Thus far, spreading or progression to malignancies has not been reported. Given that resection is curative and recurrence appears to be associated only with incomplete resection, surgical excision should be the primary aim in every case, even if it results in functional impairment.

Fasciitis ossificans is characterized by abnormal, extraskeletal ossification in inflamed fascial tissue and can be reactive to trauma or surgery, or secondary to chronic inflammation [3]. Histologically, it consists of fibroblastic connective tissue, cartilage, bone, and osteoid, distinguishing it from myositis ossificans, where active ossification is absent [4]. The origin of the connective tissue is the primary distinguishing feature. However, due to ossification, misinterpretation as osteosarcoma can occur, leading to inappropriate treatment. Ubiquitin-specific protease 6 (USP6) rearrangements have been identified as a consistent marker in nodular fasciitis, confirmed by fluorescence in situ hybridization (FISH), and may offer diagnostic assistance in differentiating fasciitis ossificans as well [5].

## Conclusion

For the first time, we report a case of fasciitis ossificans affecting the glans penis in a male individual, characterized by tumor formation mimicking penile cancer. The diagnosis was confirmed through evidence of bone formation in the specimen. Given the advanced stage of the disease and significant lower urinary tract symptoms, organ-sparing surgery did not appear to be a suitable approach. Although fasciitis ossificans of the penis is exceedingly rare, its consideration should be warranted in cases of unclear tumor formation.

## Data Availability

Patient reporting data and material are stored at the clinic’s patient documentation file. The dataset analyzed during the current study are available from the corresponding author on reasonable request.

## References

[1] Bernstein K, Lattes R. Nodular (pseudosarcomatous) fasciitis, a nonrecurrent lesion: clinicopathologic study of 134 cases. Cancer, 1982. 10.1002/1097-0142(19820415)49:8<1668::aid-cncr2820490823>3.0.co;2-9 6279273

[2] Rosenberg AE. Pseudosarcomas of soft tissue. Arch Pathol Lab Med. 2008. 10.5858/2008-132-579-POST 18384209

[3] Rozen WM, et al. Fasciitis ossificans with a radial neuropathy: a benign differential diagnosis for soft tissue sarcoma. J Clin Neurosci; 2007. 10.1016/j.jocn.2006.01.009 17240146

[4] Kempson RL (2001). Tumors of the soft tissues, Atlas of Tumor Pathology.

[5] Salib C (2020). USP6 gene rearrangement by FISH analysis in cranial fasciitis: a report of three cases. Head Neck Pathol.

